# Religiosity and perceptions about research misconduct among graduate nursing students

**DOI:** 10.1002/nop2.563

**Published:** 2020-07-17

**Authors:** Sawsan Abuhammad, Karem Alzoubi, Tareq L. Mukattash

**Affiliations:** ^1^ College of Nursing Jordan University of Science and Technology Irbid Jordan; ^2^ Department of Clinical Pharmacy Faculty of Pharmacy Jordan University of Science and Technology Irbid Jordan

**Keywords:** graduate students, Islam, perception, religiosity, research conduct

## Abstract

**Aim:**

This paper aims to study the correlation between religiosity and research misconduct in graduate nursing students and determine other factors that may have an impact on research misconduct in graduate nursing students.

**Design:**

A cross‐sectional descriptive study was used to collect data from 49 graduate nursing students.

**Method:**

The target population comprised all graduate nursing students enrolled at Jordan University of Science and Technology, Jordan. Participants who met the criteria were approached by the researcher and given a letter that provided detailed information about the study, and their informed consent was obtained. The questionnaire was consisted of three parts: a demographic datasheet, research misconduct tool and a religiosity questionnaire.

**Results:**

The results demonstrated a significant interaction between perceptions about research misconduct and one of predictors such as religiosity, where higher religiosity was associated with perceiving research misconduct as a critical issue.

## 1 INTRODUCTION

All professions and specialties require scientific research. Research improves the state of science in all fields (Lips‐Wiersma, 2002). The knowledge gained can be used towards the benefit of both researchers and society. However, researchers must have the highest standard of education and specialist and expert knowledge to be able to enhance the state of science. Graduate students in the field of nursing and other health disciplines are not only potential researchers, they are also the future healthcare providers; thus, the society needs to invest in them (Abuhammad, Hatamleh, Howard, & Ahmad, 2018; Resnik, 2011). These students need to learn how to conduct research ethically and responsibly, to meet the moral standards expected from the health profession (Noddings, 2013).They need to recognize the importance of learning and applying a code of ethics to enhance their moral judgement in conducting research.

Future nurses and other health professionals who are new to the research process need to have adequate knowledge of all the ethical and research misconduct issues that they may face as graduate nursing students. However, many graduate students in the field of health do not complete courses focused on the ethics of misconduct in areas such as authorship, reporting results, ethical aspects of animal experimentation, conflict of interest and publication(Grady et al., 2008; Marks, 2009). Furthermore, if they are exposed to the literature on research ethics, most of the articles and books discuss these issues in a philosophically narrow manner and focus on the researcher's behaviour towards an ethical dilemma (Godbold & Lees, 2013).

Religiosity has been generally associated with higher moral and ethical standards (Marks, 2009). In fact, religion was shown in some groups to be woven into everyday life, being the major factor of making distinctions between wrong and right (Koenig, 2013; Reimer‐Kirkham, 2009; Shariff & Norenzayan, 2011). Presently, there is little evidence on whether there is a relationship between practising the Islamic religion and research misconduct. Therefore, studies need to be undertaken to establish whether an increased commitment to religious practice by a researcher is associated with a decreased prevalence in practising wrong behaviours such as research misconduct. An analysis of the perceptions that future nurses hold about research misconduct will therefore along this line fill this knowledge gap. Therefore, the current study was undertaken to understand the knowledge and perceptions of research misconduct among graduate health students of a nationally representative and convenient sample of nursing graduates in a large university. The results of the current study will provide a more useful knowledge base on perceptions towards science, research misconduct and some of the influencing factors, such as religiosity, that may help in decreasing the incidence of research misconduct. It will also help educational policymakers to determine the topics that need to be covered in graduate nursing and other health programmes.

## 2 METHODS

This study used a cross‐sectional correlational design to describe the association between religiosity and research misconduct in nursing graduate student in one of Jordan's universities. A convenience sampling approach was used to recruit graduate nursing students from Jordan University of Science and Technology. Forty‐nine graduate nursing students were participated in the study.

### 2.1 Inclusion criteria

All Jordanian female and male graduate nursing students who could read and write Arabic and were studying at from Jordan University of Science and Technology at the time of study were eligible to participate in the research. Participants were excluded if not able to read and write in Arabic language and not currently enrolled to nursing graduate programme.

### 2.2 Procedure

The researchers contacted the deanship of research in Jordan University of Science and Technology to inform them about the purpose and nature of the study. The investigators explained that they wanted to undertake a study to understand whether there was a significant correlation between Religiosity and research misconduct. The university was advised of the strict confidentiality standards that would be put in place by the researchers to protect both the student and university data. After the investigators had received permission to conduct the research and to use student and university data, they provided this information to the Institutional Review Board (IRB) at Jordan University of Science and Technology for authorization of the research. After approval was granted, the researchers provided the research study dissemination instructions and questionnaire packets to lecturers/ members of the faculty of nursing and then started collecting data.

The questionnaire that was developed for this study consisted of three parts: a demographic datasheet, a head of department questionnaire on research misconduct and a religiosity questionnaire. Consenting was waived by the IRB. Answering the survey was considered as approval and consenting for the study. This study was approved by the IRB of Jordan University of Science and Technology. Participants who met the criteria were approached by the researcher and given a letter that provided detailed information about the purpose of the study. It stated that the researcher would use the information obtained from the study to help practitioners and people in the community. It also stated that the researcher would maintain the privacy of the graduate nursing students' information and would not use any information in presentations or publications in a way that could lead to the identification of any participants. It also confirmed that the principal investigator and agents of the Office for Human Research Protection and Jordan University of Science and Technology had appropriate regulatory oversight of the study.

The questionnaire was administered electronically by sending it to graduate nursing students in the nursing faculty at Jordan University of Science and Technology via WhatsApp or Facebook. The questionnaire was tested and found to be easy to complete and approximately 20 min were required to complete it. The researchers provided a phone number and email on the cover page of the electronic questionnaire so that students could contact the researchers to clarify any issues they encountered in relation to the completion of the questionnaire.

### 2.3 Demographic data

The demographic datasheet consisted of questions about age, gender, marital status, graduate degree, mother's education, father's education and intent to work as a researcher.

### 2.4 Perception of research misconduct questionnaire

This instrument was designed to measure perceptions about research misconduct (Sailor, 1998). It consisted of 44 items that were measured by using a Likert scale ranging from 1 (strongly disagree)–7 (strongly agree). It encompassed all the types of misconduct discussed in the literature from less serious to outright fraud to serious incidents that constitute misconduct. The instrument focused on four categories of ethical problem: personal integrity (14 items), methodology (six items), data analysis (seven items) and publication (17 items). The scale consists of 44 items responses ranging from not serious (1)–very serious (7) to measure negative‐to‐positive variance The items on personal integrity included asking for funds to support work already done; not informing human subjects correctly and adequately; and false claims made for grant proposals. Those on methodology covered issues such as fabrication of data; failure to have data available when requested; and violation of methodological concerns). Some of the items on data analysis included selective deletion of “outlying” data points; honest errors; and incompetent data analysis.

Finally, the items on publication covered issues such as not disclosing weaknesses in data; not disclosing weaknesses in research design; and not presenting results that contradict one's previous research. Cronbach's alpha coefficients for each of these subscales were as follows: 0.84 for personal integrity, 0.70 for methodology, 0.72 for data analysis and 0.89 for publication. Cronbach's alpha for the item scale of the whole questionnaire was 0.94, which means that it had a high degree of internal consistency. Correlations between subscales ranged from 0.58–0.77 (Waardenburg & Waardenburg, 2002).

### 2.5 Religiosity questionnaire

This instrument consisted of five subscales that were specifically applied to the religion of Islam: basic religiosity, central duties, religious experience, religious knowledge and orthopraxis. The items for belief consisted of belief in the existence of Allah; belief in Allah's book (Qur'an); and belief in the presence of Jinn and angels (El‐Menouar, 2014). The items for central duties in Islam consisted of the five pillars of Islam: Al‐Shahadah, prayer (five *salat* per day), almsgiving (*zakat*), fasting (*swam*) and pilgrimage (*hajj*). The devotion subscale had additional subscales that consisted of frequency of personal prayer to Allah and frequency of recitation of the *Basmala*. Religious experience in Islam links specific events to some purposeful supernatural powers, which refers to the feeling of Allah's presence and communicating with or receiving a certain type of communication from Allah. Religious knowledge for Muslims means having the required knowledge of Islam as a religion and a lifestyle. The Qur'an and the Prophet Mohammad's deeds are the main sources learned in relation to the notion that Islam regulates the daily aspects of Muslim life (Al‐Shatanawi, 2018). Orthopraxis or the notion of consequences to actions arises from the level of internal belief of the Muslim faithful in the 71 dichotomous consequences of certain deeds: heaven or hell, happiness or sadness, punishment or reward as explicated in a previous study by El‐Menouar (2014). The scale consists of 30‐item responses ranging from never (1)–always (4) to measure negative‐to‐positive variance. Cronbach's alpha for the five dimensions were as follows: basic religiosity = 80, central duties = 0.88, religious experience = 0.87, religious knowledge = 0.86 and orthopraxis 0.76.

### 2.6 Data analysis

Following data collection, responses were coded and entered into a customized database in SPSS version 24 for subsequent statistical analyses. All the data were stored with a coded subject identification number, and any link between the coded data and the identifying information was removed after the data had been analysed. The obtained data were analysed, aggregated and used to produce the results of the study. No names or other identifying information were attached to any data. Correlation test was conducted between perception towards research misconduct and religiosity. Furthermore, multiple regressions were conducted to determine other factor that may detect perception towards research miscon).

## 3 RESULTS

### 3.1 Descriptive statistics

Among the 49 participants, 41 (84%) were female and eight (16%) male. The age of both gender groups ranged from 23–40 years, and the mean age was 28.2 years. The religious belief of all the participants was Islam. No participants were excluded, since the survey just suitable for Muslims. Among the participants, 43 (88%) expected to work as a researcher, whereas 6 (12%) did not. As regards the marital status of the participants, 28 (57%) were single and 21 (43%) were married. Income was ranged between 2,100 JD (3,200$) to more than 4,900 JD (7,000 $). The mean of income was 3,600 JD (5,200$) annually and *SD* (9.53). Most of the students were in their second year in graduate studies 30 (60%). Most of the fathers of the participants had a bachelor's level of education (*N* = 21, 42.9%), followed by a primary or secondary (*N* = 15, 30.6%), associate (*N* = 5, 10.2%) and graduate (*N* = 8, 16.3%) level of education. On the other hand, most of the mothers of the participants had an education at the primary or secondary level (*N* = 21, 42.9%), followed by bachelor's (*N* = 14, 28.6%), associate (*N* = 12, 24.5%) and master or PhD (*N* = 2, 4.1%).

Next, Pearson's correlation test was conducted to determine whether there was a correlation between religiosity and perceptions about research misconduct. Religiosity scale consists of 30 items with the score ranges from 30–120. Increasing score means more religiosity. The mean score of religiosity was 103.4 and *SD* (16.44). The perception of misconduct scale consists from 44 items with the score ranges from 44–308. The mean score of perception towards research misconduct was 235.7 (*SD* 56.82). Increasing score means more seriousness towards research misconduct as harmful and avoid it. The result revealed that religiosity correlated strongly with research misconduct (*r* ^2^ = 0.55, *p *= .01).

Then, multiple regressions were carried out to determine whether there were other predictors of perceptions about research misconduct. The regression model was significant (*F* = 18.89, *p *< .01), which means there is a factor or more essential in predicting perceptions about any type of research misconduct. Multiple regression model of variables that are statistically significantly associated factor with perception towards research misconduct found that only age was statistically significant (*p* < .05). It can be noticed that a significant interaction only exists between perceptions about research misconduct and age (*t* = 1.87, *p *= .05, *SE* = 2.37). It is important to note that all of the demographic variables (age, gender, specialty area, marital status, education level, mother's education, father's education and intent to work as a researcher) were not significant as their *p* values were all higher than .05. See Table 1.

**TABLE 1:** Multiple regressions for the relationship between demographic data and research misconduct

Model 1	Unstandardized coefficients		Standardized coefficients	*t*	Sig.
	*B*	*SE*	Beta		
(Constant)	−192.027	109.320		−1.757	0.087
Intent to work as a researcher	31.920	31.144	0.153	1.025	0.312
Age	4.418	2.365	0.293	1.868	0.049
Gender	13.638	26.760	0.073	0.510	0.613
Marital status	−3.077	19.569	−0.022	−0.157	0.876
Income	11.489	7.918	0.203	1.451	0.155
Father's education	3.891	9.312	0.060	0.418	0.679
Mother's education	−1.632	11.960	−0.021	−0.136	0.892
Sum religiosity	1.651	0.511	0.461	3.228	0.003

## 4 DISCUSSION

The importance of addressing ethical and moral challenges in graduate programmes, particularly in health professions such as nursing, has been subject of some interesting discussions in the literature in recent years. However, a massive gap still exists in the literature regarding how religiosity variables relate to perceptions about research misconduct. In fact, there are studies that portray correlation between the perception and religiosity of research misconduct (Sailor, 1998). As the analysis of research misconduct as a social construct is a relatively new area, it is difficult to draw firm conclusions from the results of the current analysis or to compare the results with those in other related works. Nevertheless, they seem to suggest that the Islamic religion could possibly have an impact on the way issues regarding ethics, particularly the types of ethics addressed in this study, are dealt with by the academic community.

The results of our study revealed that religiosity correlated strongly with research misconduct (*r* ^2^ = 0.55, *p *= .01). Our results were congruent with previous studies. Previous research indicates that those who state that they have a certain level of religiousness are less inclined to undertake immoral or risky behaviours and are more likely to have a higher responsibility level. For example, Laurin, Kay, and Fitzsimons (2012) stated that stimulating the concept of God (i.e. those who have an intrinsic religiosity orientation) in people's mind can lead to a greater resistance to immoral and unethical behaviours one of them is misconduct. “Thus, religion variables may predict greater temptation resistance in moral and religious domains in part because they also predict more frequent activation of the God concept” (p. 14). In another study, Randolph‐Seng and Nielsen (2007) assured that the presence of religious representations may cause people, not just to refuse unethical behaviours but also to behave more honestly. There is a focus of Islamic religion in the context of social construct, on the concepts behind the modification of behaviour. An assumption was reached regarding how behaviour modification and adherence are related to the holding of religious principles, which would be the most relevant variable to the research misconduct in particular (Vieno, Nation, Perkins, & Santinello, 2007). Different cultures have benefited from religion for many centuries (Davis, 2003).

In the event that the literature above is considered valid and misconduct behaviours are engaged, at this point, previous literature indicated the fact that a relationship still exists since no result is shown. Among all 192 students, only 1 per cent of them reported long‐term, consistent, performing unethical behaviour such as research misconduct. From the literature, more than 77 per cent of students stated that they had committed some form of research misconduct, even in their school days. Yet, the school investigations of such misconduct were significantly lower. Even though a correlational study does not ascertain any causality, an assumption of the religious‐based curriculum is considered, factually, having a few impacts on research misconduct; this is a quandary that can be found alongside the research study results. Just as it is hard to ascertain causality in this study, it is important to assume that something regarding this study setting exists, which makes it different from its contemporaries. It is not an entirely stretched one to ascertain that something regarding the setting made them encounter less bullying as opposed to other schools with the same makeup and size (Davis, 2003; Hugen & Venema, 2009; Parboteeah, Hoegl, & Cullen, 2008). Probably, it is the actual values that meant the Islamic religion of the school has assisted in viably getting rid of research misconduct nowadays (Parboteeah et al., 2008).

Values like these have provided a base and even some paradigms in the health organizations, particularly in nursing organizations, alongside the perspective of achievement as well as highly sufficient development of the individual potential of individuals living a professional life. Nowadays, organizations are viewed as live systems of people and, therefore, there is a growing realization that human capital is ready to act, not just on the body and mind dimensions, but also on the religious dimension (Jang, Johnson, Hays, Hallett, & Duwe, 2018; Shakoor et al., 2012). Providing a response to the present demands of health institutions, the attitudinal commitment ethics permeated conduct, which the nurses demanded. There is a relationship between a dimension of attitudinal commitment and interiority (Abuhammad, Alnatour, & Howard, 2020). This idea shows that the work of nurses is based on the premise of their personal acceptance of interior reality as well as other individuals' acceptance and of being capable of feeling compassionate, comprehending and feeling generous.

## 5 CONCLUSION

From the results, it is clear that the perceptions that graduate nursing students hold about research misconduct are associated with religiosity. The measurements in this study were based on a religiosity subscale as well as its subscales, leading to the conclusion that there is a need for more research to describe the factors related to the perception of research misconduct. More knowledge on the influence of socio‐demographic attitudes, values, beliefs and other variables is required.

## CONFLICT OF INTEREST

The authors have no funding or conflict of interest to disclose. All authors participated in all research steps.
